# Gene Expression Based Leukemia Sub-Classification Using Committee Neural Networks

**DOI:** 10.4137/bbi.s2908

**Published:** 2009-09-03

**Authors:** Mihir S. Sewak, Narender P. Reddy, Zhong-Hui Duan

**Affiliations:** 1 Department of Biomedical Engineering, University of Akron, Akron, OH 44325-0302; 2 Department of Biomedical Engineering and Integrated Bioscience Program, University of Akron, Akron, OH 44325-0302; 3 Department of Computer Science and Integrated Bioscience Program, University of Akron, Akron, OH 44325-4003. Email: duan@uakron.edu

**Keywords:** leukemia cancer, neural networks, microarray, gene selection, sample classification

## Abstract

Analysis of gene expression data provides an objective and efficient technique for sub-classification of leukemia. The purpose of the present study was to design a committee neural networks based classification systems to subcategorize leukemia gene expression data. In the study, a binary classification system was considered to differentiate acute lymphoblastic leukemia from acute myeloid leukemia. A ternary classification system which classifies leukemia expression data into three subclasses including B-cell acute lymphoblastic leukemia, T-cell acute lymphoblastic leukemia and acute myeloid leukemia was also developed. In each classification system gene expression profiles of leukemia patients were first subjected to a sequence of simple preprocessing steps. This resulted in filtering out approximately 95 percent of the non-informative genes. The remaining 5 percent of the informative genes were used to train a set of artificial neural networks with different parameters and architectures. The networks that gave the best results during initial testing were recruited into a committee. The committee decision was by majority voting. The committee neural network system was later evaluated using data not used in training. The binary classification system classified microarray gene expression profiles into two categories with 100 percent accuracy and the ternary system correctly predicted the three subclasses of leukemia in over 97 percent of the cases.

## Introduction

Leukemia affects more than 44000 individuals each year in the United States alone, and it has one of the top mortality rates among different types of cancer.^1^ Leukemia is the cancer of the blood. It belongs to a broad array of diseases commonly referenced as hematological malignancies. If the disease occurs in the lymphocyte forming marrow cells then it is called lymphocytic or lymphoblastic. T-cell acute lymphoblastic leukemia (T-cell ALL) is observed in 15 percent of lymphoblastic leukemia patients whereas acute B-cell lymphoblastic leukemia (B-cell ALL) affects 85 percent of all the acute lymphoblastic leukemia (ALL) patients. If the acute disease occurs in the bone marrow cells that form the red blood cells, white blood cells or platelets then the term acute myelogenous or acute myeloid leukemia (AML) is employed. There is a significant need to detect and subcategorize the disease at its inception. Also, accurate subtype classification is absolutely vital as the treatment protocol varies significantly from one subtype to the other.

Traditionally, leukemia cells have been categorized considering their morphological appearance. Highly skilled resources are needed to identify the differences between tumor cells. The process can be tedious, time intensive and highly expensive. It is interesting to note that even with the adequate availability of the necessary resources the technique is not foolproof. Cells can appear similar morphologically but respond very differently to cytotoxic drugs and therapy.^2^ These limitations of the traditional technique led to a need to identify other parameters which could be used as a basis for cell categorization. Gene expression data may provide useful information for sub-classification studies. DNA microarrays have played an important role in monitoring gene expression data of thousands of genes at the same time. It is possible to determine the comparative levels of expression of genes in normal cells against abnormal cells. Study of expression levels can lead to helpful insights in making classification decisions based on gene signature of cells under question.

Recently, several studies have reported utilization of machine learning techniques for differential classification of leukemia. The first study on leukemia subtype classification using microarray data was performed by Golub et al.^3^ The study built a binary classification system in order to automate classification of leukemia into its primary subclasses. It identified a list of genes whose expression levels correlated with the class vector, which was constructed based on the known subtypes of the samples. This list of genes was considered as informative genes. The sample classification was then performed using a neighborhood analysis method based on the information provided by the informative genes. Their study verified the conjecture that there were a set of genes whose expression pattern was strongly correlated with the class distinction to be predicted and this set of informative genes can be used for sample classifications. Strong predictions for 29 of the 34 test samples with 100% accuracy were achieved. In addition to the supervised classification problem, an automatic class discovery method, self organizing maps method, was also explored in the study. The study concluded that it was possible to classification cancer subtypes based solely on gene expression patterns. Mallick et al^4^ used several Bayesian classification techniques for leukemia sub-categorization (ALL and AML). Antonov et al^5^ used a maximal margin linear programming approach. They utilized the information content of 185 genes to obtain 100 percent prediction accuracy in classification of leukemia into two subclasses. Several researchers have used variations of support vector machine based algorithms to tackle the leukemia sub-classification problem.^6^–^8^ Several other investigators^9^,^10^ have used neural networks for cancer classification problems. Berrar et al^9^ developed a multi-classification system that took into account the subclasses of ALL and AML. They used probabilistic neural networks to classify leukemia into eight classes. An accuracy of 62 percent was achieved. The performance of a neural network depends on several factors including the initial random weights, the training data, the activation function used, and the structure of the network including the number of hidden layer neurons, etc.^11^–^14^ Machine learning theory points to the fact that the reliability of a machine learning based classification system significantly depends upon the nature of the input data, the amount of training nurtured to the classification system and the ability of the algorithm to adapt to the incoming data. The confidence and the reliability of the classification system can be improved by using an ensemble of techniques. Reddy et al^11^,^12^ Palreddy et al^13^ and Das et al^14^ have developed and evaluated the technique of committee neural networks. They found significant improvement in the prediction performance with committee networks when compared to individual networks. This technique yields a confirmed classification or misclassification with more reliability. A similar technique has been used to classify cancer samples based on microarray data by.^15^,^16^ In the study by Liu et al^15^ an advanced combinational feature selection method in conjunction with ensemble neural networks was introduced. They found that the method generally improves the accuracy and robustness of sample classification. This study uses a simple feature selection method,^17^ non-parametric t-test for the binary system and analysis of variance for the ternary system, and explores the accuracy and stability of a system of committee neural networks when it is used to sub-classification of gene expression data from leukemia patients.

## Methdology

### Data

This study used the leukemia gene expression data collected by Golub et al.^3^ This dataset comprised of 72 bone marrow samples of which 38 were B-cell ALL, 9 were T-cell ALL and 25 were AML samples. Each profile was made up of quantitative expression levels for 7129 probes. In the original study of this set of microarray gene expression data, the data from 37 patients was used for training and the data from the remaining 35 patients was used in testing. In this study, the testing dataset was divided into two subsets of 8 and 27 samples each. The first set of 8 samples was used in initial testing and remaining 27 samples were used in final testing.

### Data preprocessing

The training dataset consisted of gene expression profiles for 37 patients. Each profile comprised of 7129 gene expression values. The preprocessing was carried out on the same lines of Dudoit et al.^18^ Endogenous control genes were eliminated from consideration. Genes with “*absent*” calls across samples were eliminated from consideration. Genes with less than 2.5 fold change across samples were eliminated from consideration. Genes with expression values of less than 20 and greater than 16000 were thresholded to 20 and 16000 respectively. The remaining gene expressions were normalized so that the expression levels are in the range of −1 to 1. For the binary system, non-parametric t-test was used to select informative genes. The genes were resorted according to increasing p-values. Highly informative genes can be obtained at the top of the list. In case of the ternary system, an analysis of variance (ANOVA) was carried out across three classes for each gene set.

### Training of neural networks

It had been reported that neural network classifiers based on about top 100 genes outperformed classifiers with significantly less or more genes.^10^ In this study, to ensure most of informative genes are included in building the classifiers, the top 250 genes from the preprocessed list were considered in the training of several neural networks. The 250 genes were then divided into ten groups of 25 genes each. Each of these gene groups were used to train several multilayer feed forward fully connected neural networks. Each of these networks had 25 input nodes corresponding to each gene expression and an output node corresponding to a leukemia subclass. For the binary system, the two output nodes represent the subclasses ALL and AML. In case of the ternary system, they are T-ALL, B-ALL and AML. The values obtained at each output node were converted to binary format. The outputs are in the range of 0 to 1. When the output is “1”, it indicates the test sample belong to the class. “0” indicates the sample does not belong to the class. It is seldom the case that a test sample gives an exact “0” or an exact “1” at the output layer. Hence we forced any output greater than 0.7 to “1” and floored any output lesser than 0.7 to “0”. Previous studies have used values between 0.6 and 0.95 as the thresholding levels.^11^–^14^ The parameters used in this study were based on these values and finalized through trial and error. The exact thresholds varied depending upon the nature of study. Several networks were trained with each of the ten datasets. These networks differed from one another in the initial weights, number of hidden input layers (2 to 3 hidden layers), and number of neurons in each hidden layer (12 to 26 hidden layer neurons). It was observed that the networks converged very efficiently when a hyperbolic tangent sigmoid function was used for the hidden layers and a log sigmoid function was used for the output layer. All the neural networks were trained using the Levenberg-Marquardt function in MATLAB.^19^ The error goals were set to 1e-10 and the number of epochs was set to 1000.

### Recruitment of the neural network committee

The trained networks were subjected to initial validation. The initial validation set comprised of eight samples encompassing all subclasses of leukemia. Networks that performed best in evaluating the initial validation dataset were considered in the recruitment of a neural network committee. The best networks were recruited into a committee based classification system. The majority opinion of the individual members of the committee formed the overall decision.

### Evaluation of the committee

**Figure 1.**
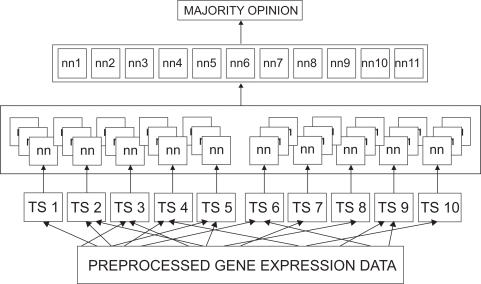
Architectural diagram of the Committee Neural Network System: The 250 gene expression data was divided into ten sets of 25 each. Several neural networks were trained using each of these sets. Each network has three output nodes corresponding to each classification (T-ALL, B-ALL, and AML). The best performing 11 networks were recruited into a committee. The committee decision was by majority opinion.

The performance of the recruited committee was evaluated using a fresh dataset of 27 samples. These consisted of 17 B-cell ALL, 2 T-cell ALL and 8 AML samples. The samples of the final evaluation dataset were different from those present in the training dataset and the initial validation dataset. Figure 1 presents the architectural diagram of the designed ternary classification system.

## Results

### Data reprocessing

**Figure 2.**
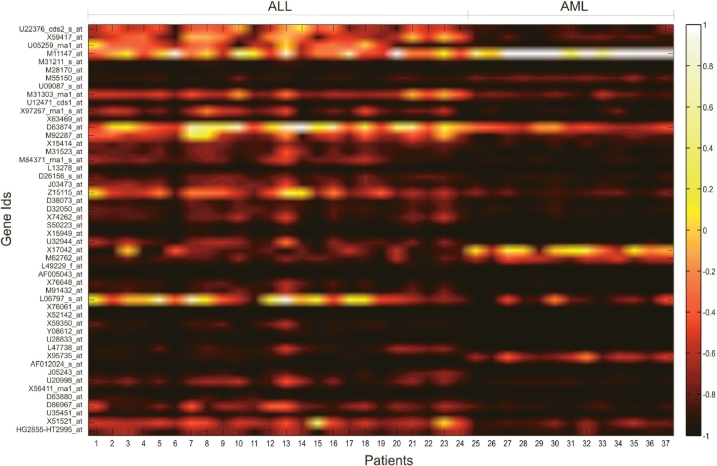
Expression intensity values of top 50 genes for the binary classification system are scaled to a ‘*hot*’ color map. The x-axis displays the patients clubbed according to disease while the y axis shows informative genes.

**Figure 3.**
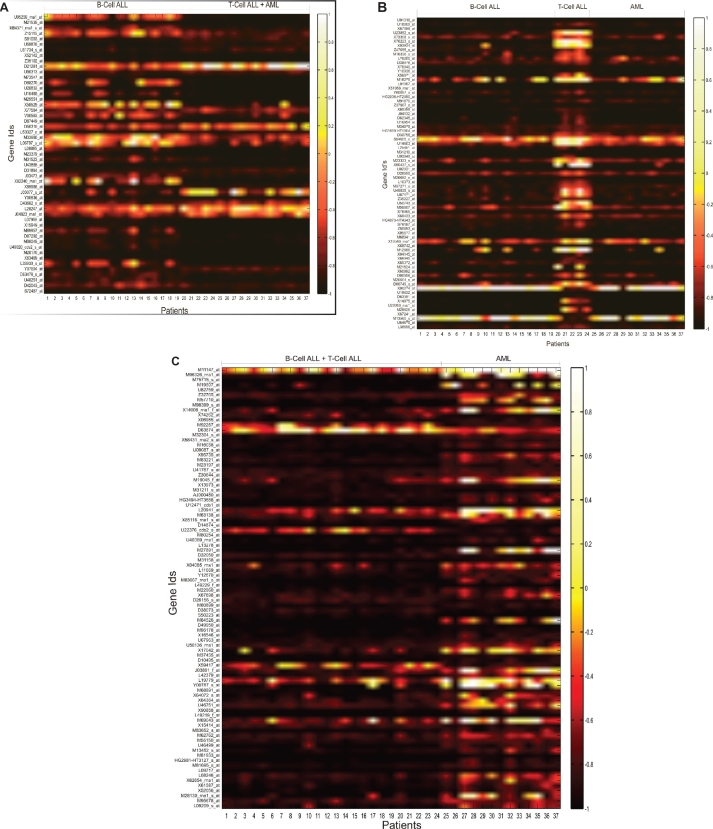
Expression intensity values of the top most informative genes for the ternary classification system are scaled to a *“hot”* color map. The x-axis displays the patients grouped according to the disease while the y-axis shows informative genes. Figure 3a shows genes that differentiate B-ALLs from T-ALLs and AMLs. Figure 3b shows genes that differentiate T-ALLs from B-ALLs and AMLs. Figure 3c shows genes that differentiate AMLs from B-ALLs and T-ALLs.

The data preprocessing steps led to filtration of 2334 genes which were deemed non-informative and eliminated from consideration. After the genes were ordered according to increasing p-values, the genes at the top of the list yielded a high level of differential information. The top 250 genes from this list were considered for preparation of parameter sets for the neural networks. Figure 2 shows the gene expression values of the top 50 genes for the binary classification system. In the figure, the actual values of expressions were scaled to a full range of a hot color map and the intensity values were plotted. Distinct division between the two groups of data can be seen in the Figure. In case of the ternary system, of the 250 genes 51 genes showed differential expression for B-cell ALLs, 88 genes showed differential expression for T-cell ALLs and 73 genes showed differential expression for AMLs. The intensity values are plotted in the form of heat maps in Figures 3a, b, and c.

### Performance of the committee

For the binary classification system, the preprocessed data was used to train thirty six neural networks. The trained networks were initially tested against an initial validation set comprising of eight samples. The worst case performance was from a network which correctly predicted only four of the eight samples of the initial validation set. The best case performance was achieved by one network which rightly classified all 8 samples. Five top performing networks were recruited into a committee. The committee gave 100 percent classification accuracy for the initial validation set as shown in Table 1. This committee was then used for validating a fresh set of data comprising of 27 samples. Of the individual networks of the committee, the best case performance was obtained by three networks which rightly predicted the class in all the 27 cases. The worst case performance was obtained by one network which predicted the right class 24 out of 27 times (Table 2). However, the committee decision with majority opinion correctly classified the data in 100 percent of the cases (Table 3). Since neither the initial validation nor final validation datasets was used for training purposes, the combined accuracy achieved by the system for 35 samples was 100 percent.

Similarly, in case of the three class problem, the preprocessed data was divided into ten groups of 25 genes each. Each of these groups was used to train around 10 to 12 neural networks. A total of 115 networks were trained in this manner. Each of the trained networks was validated against the initial validation datasets. The worst case performance was from 5 networks which correctly predicted only four of the eight samples of the initial validation set. The best case performance was achieved by 20 networks which rightly classified all 8 samples. Networks which accurately predicted at least 7 out of the 8 test cases were considered. A committee of 11 networks was formed and evaluated against the final testing dataset of 27 samples. Table 4 presents six classification results that explain the majority voting technique of a neural network committee. The values in bold indicate misclassifications. The actual classes were obtained from lab results and were downloaded from the website of the Broad Institute.

The recruited committee gave 100 percent classification accuracy for the initial validation set (Table 5). The recruited committee was further validated using a fresh set of data comprising of 27 samples, it correctly predicted 26 out of the 27 final validation cases to yield a prediction accuracy of 96.29 percent (Table 6). Of the individual networks of the committee, the best case performance was obtained by three networks which correctly predicted the class in 26 out of the 27 cases. The worst case performance was obtained by one network which rightly predicted the class 20 out of 27 times. Since neither of the initial validation and final validation datasets was used for training purposes, the combined accuracy achieved by the system for 35 samples was 97.14 percent (Table 7). The confusion matrix for the system is presented in Table 8. It gives a class-wise distribution of accuracy of the individual networks and that of the committee.

## Discussion

The present study represents an application of committee neural networks for leukemia classification using microarray gene expression data. The classification systems required a very simple preprocessing procedure and gave an accuracy of 100 percent for a two class classification problem (Table 3) and 97.14 percent for a three class classification problem as it failed to classify 1 out of 35 validation sets (Table 7). For both systems, only one network from the individual committees managed to achieve an equivalent accuracy as that of the committees. Furthermore, considering the heuristic nature of machine learning algorithms, the committee decision provided a highly reliable result with more confidence than the individual networks.

The present study represents a step forward in sub-classification of Leukemia. Several researchers have worked on the Leukemia dataset as a two class problem and obtained accuracies in the range of 80 to 100 percent in their efforts. Golub et al^3^ classified leukemia data into ALL and AML. They used a nearest neighbor analysis technique to identify 1100 genes which provided differential information for the two sub-classes. Their technique correctly predicted 29 out of 34 test cases to yield an accuracy of only 85 percent. Mallick et al^4^ constructed binary classification models for differential classification of different cancers, including leukemia. The Bayesian classifiers that they developed were based on the Reproducing Kernel Hilbert Space (RKHS) approach. They constructed multiple models for their classification purposes and obtained accuracies in the range of 85 percent to 97 percent. Berrar et al^9^ worked on multi-classification of the leukemia dataset. They designed a probabilistic neural networks based classifier to subcategorize ALLs and AMLs into six sub-classes with a prediction accuracy of 62 percent. In the present study, the accuracy of the two-class classifier was 100 percent. In case of the three-class classifier, the classification accuracy was 97.14 percent. We note that the accuracy level was obtained based on the available number of samples in the three subclasses of leukemia cancer. The sub-categories of AML were not considered individually in the study, because there were not adequate samples to have representation in the training, initial and final test datasets.

The present study developed a technique of using different data parameters to train different networks. The committee consisted of member networks trained with different data. The size of the informative gene sets has varied from less than ten to greater than hundred. The drawback of using small gene sets in designing classification systems is that the decision is based on a very small informative set thus raising questions on the reliability of the system. Since the expression of a gene is dependent on a lot of conditions some known while others unknown, it is never advisable to make a decision based on a very small gene expression set. Utilization of large gene expression sets, suffer from other problems. Although, it ensures that enough information is available to base a decision upon, the drawback of using large datasets is the increased complexity of the machine learning algorithms and, the high probability of numerical instability in the system. The present study utilized the positives of both approaches. By incorporating several processing elements with small input parameter sets (25 inputs per processing element) each, we kept the mathematical complexity as low as possible. At the same time, by using 250 informative genes, our classification system based its judgment on a sufficiently large informative set. It was observed that the 250th most informative gene had a p-value between classes less than 0.001 thus giving an idea about the level of information that the genes imparted to the system.

The preprocessed set comprised of the top 250 highly informative genes. It is known that genes interact with each other through biological networks such as regulatory networks and signaling networks. To identify the interconnection and reduce the redundant genes in the classification models are yet to be done. In this study, we relied on the neural networks with varying architectures to interpret the information content of each gene. More than 100 networks were trained using the sample sets of 25 genes each. The architectures of the individual networks varied in the number of hidden layers, the hidden layer neurons, the transfer functions and the learning functions. Although the training experiments were scheduled to terminate either upon reaching 1000 epochs or an error goal of 1e–17, it was observed that network convergence was obtained well before the limiting conditions were reached. The networks gave a consistent performance in terms of training time and accuracy for one to two hidden layers comprising of 14 to 25 hidden neurons. The performance became inconsistent when these parameters were modified. Intermediate validation was carried out on a set of eight samples.

For the two class classification problem, the committee neural networks provided 100 percent accuracy for both initial and final validation data sets. In case of the three class problem, the initial validation gave 100 percent accuracy. However, the classifier misclassified a T-cell ALL sample as an AML when tested against the fresh validation set. One reason for this misclassification was the limited number of samples in the training set. Although the neural network converged to zero, only five samples of T-cell ALLs were present in the training set. Availability of more samples to represent the class of T-cell ALLs may have improved the prediction accuracy.

Reddy et al^11^,^12^ Das et al^14^ and Palreddy et al^13^ have developed and used committee networks for classification of swallow acceleration signals and speech signals. However, in all theses studies, the same input vector was used to train a series of networks. The current study used different sets of input parameters to train different neural networks. This technique was analogous to the concept of a jury decision with members having widely differing backgrounds.

## Conclusion

The gene expression profiles, each more than 7000 genes, were successfully processed to identify a subset of 250 genes that could distinguish the classes from each other. A committee neural network system was designed to classify leukemia into ALL and AML using the microarray gene expression data. The classification accuracy was 100 percent. A committee neural network system for three class classification was also developed to classify leukemia into T-cell ALL, B-cell ALL and AML using the expression data. The committee members were trained with different input parameters. The committee, through majority voting, correctly classified a total of 34 of the 35 validation data sets, yielding an accuracy of 97.14 percent for the three class classification problem.

## Figures and Tables

**Table 1.** Performance of the recruited committee for the two class system on the initial validation dataset.

**Network**	**NN1**	**NN2**	**NN3**	**NN4**	**NN5**	**Committee result**
Correct classification out of 8 samples presented	7	8	7	7	7	8
Accuracy	87.5	100	87.5	87.5	87.5	100

**Table 2.** Performance of the recruited committee for the two class system on the final validation dataset.

**Network**	**NN1**	**NN2**	**NN3**	**NN4**	**NN5**	**Committee result**
Correct classification out of 27 samples presented	24	27	25	27	27	27
Accuracy	88.9	100	92.6	100	100	100

**Table 3.** The overall performance of the recruited committee for the binary classification system.

**Network**	**NN1**	**NN2**	**NN3**	**NN4**	**NN5**	**Committee result**
Correct classification out of 35 samples	31	35	32	34	34	35
Accuracy	88.6	100	91.4	97.1	97.1	100

**Table 4.** Sample results to demonstrate the working of a committee system.

	**Samples**					
	**1**	**2**	**3**	**4**	**5**	**6**
NN1	B-ALL	B-ALL	**B-ALL**	**NC**	AML	AML
NN2	B-ALL	**NC**	**AML**	**AML**	AML	**NC**
NN3	B-ALL	B-ALL	**AML**	**AML**	AML	AML
NN4	B-ALL	B-ALL	**AML**	**AML**	AML	**B-ALL**
NN5	B-ALL	B-ALL	**AML**	T-ALL	AML	AML
NN6	B-ALL	B-ALL	**AML**	T-ALL	AML	AML
NN7	B-ALL	B-ALL	**AML**	T-ALL	AML	AML
NN8	B-ALL	**NC**	T-ALL	T-ALL	**NC**	AML
NN9	B-ALL	B-ALL	T-ALL	T-ALL	**B-ALL**	**B-ALL**
NN10	B-ALL	B-ALL	T-ALL	T-ALL	**B-ALL**	**B-ALL**
NN11	B-ALL	B-ALL	T-ALL	T-ALL	**B-ALL**	**B-ALL**
Committee decision	B-ALL	B-ALL	**AML**	T-ALL	AML	AML
Actual class	B-ALL	B-ALL	T-ALL	T-ALL	AML	AML

**Table 5.** Performance of the recruited committee for the three class system on the initial validation dataset.

**Network**	**NN1**	**NN2**	**NN3**	**NN4**	**NN5**	**NN6**	**NN7**	**NN8**	**NN9**	**NN10**	**NN11**	**Committee result**
Correct classification out of 8 samples presented	7	7	7	7	7	8	8	8	8	8	8	8
Accuracy	87.5	87.5	87.5	87.5	87.5	100	100	100	100	100	100	100

**Table 6.** Performance of the recruited committee for the three class system on the final validation dataset.

**Network**	**NN1**	**NN2**	**NN3**	**NN4**	**NN5**	**NN6**	**NN7**	**NN8**	**NN9**	**NN10**	**NN11**	**Committee result**
Correct classification out of 27 samples presented	26	20	26	23	25	25	25	26	24	23	23	26
Accuracy	96.3	74.1	96.3	85.2	92.6	92.6	92.6	96.3	88.9	85.2	85.2	96.3

**Table 7.** The overall performance of the recruited committee for the ternary classification system.

**Network**	**NN1**	**NN2**	**NN3**	**NN4**	**NN5**	**NN6**	**NN7**	**NN8**	**NN9**	**NN10**	**NN11**	**Committee result**
Correct classification out of 35 samples	33	27	33	30	32	33	33	34	32	31	31	34
Accuracy	94.3	77.1	94.3	85.7	91.4	94.3	94.3	97.1	91.4	88.6	88.6	97.14

**Table 8.** Confusion matrix for the three class system.

		**Actual**			
		**B-ALL**	**T-ALL**	**AML**	∑
Predicted	B-ALL	19	0	0	19
	T-ALL	0	3	0	3
	AML	0	1	12	13
	∑	19	4	12	35
Sensitivity		100	75	100	
Specificity		100	97.14	100	
